# Supplementary material for: Direct space–time manipulation mechanism for spatio-temporal coupling of ultrafast light field
Source: Nat Commun. 2024 Mar 18;15:2416. doi: 10.1038/s41467-024-46802-x (PMC10948815; doi:10.1038/s41467-024-46802-x)
Supplement: Supplementary file 1 — Supplementary Information [file 41467_2024_46802_MOESM1_ESM.pdf]

Supplementary material: Direct space-time manipulation mechanism for spatiotemporal coupling of ultrafast light field

Part I: Principle of the log-polar-to-Cartesian transformation

The log-polar-to-Cartesian transformation includes an optical lens, a geometric transformation element (GTE) and the phase compensation element (PCE). GTE and PCE are set at the front and rear focal planes of the lens, respectively. The phase functions are designed as

$$\phi_{GTE}(x,y)=\frac{-\omega_0 ab}{cf}\exp\left(-\frac{x}{a}\right)\cos\left(\frac{y}{a}\right) \tag{S1a}$$

for GTE, and

$$\phi_{PCE}(u,v)=\frac{\omega_0 a}{cf}\left[v\cdot\arctan\left(\frac{v}{u}\right)-u\cdot\ln\left(\frac{\sqrt{u^2+v^2}}{b}\right)+u\right] \tag{S1b}$$

for PCE. Here, ω_0 and c stand for the central angular frequency and speed of light in air. a and b are the size coefficients, while (x, y) and (u, v) represent the spatial coordinates at the front and rear focal planes of the lens by the relations: $u=b\cdot\exp(-x/a)\cdot\cos(y/a)$ and $v=b\cdot\exp(-x/a)\cdot\sin(y/a)$. As it is a conformal transformation [1], by exchanging the positions of the GTE and PCE phase plates, it will work as an inverse transformation, Cartesian-to-log-polar-transformation, which has been used to sort vortex beams [2].

Suppose that the incident pulse with a tilted pulse-front is

$$E_{PFT}(x,y,t)=A\cdot\exp\left(-\frac{x^2}{r_x^2}-\frac{y^2}{r_y^2}\right)\cdot\exp\left[-\alpha(t-\eta y)^2\right]\cdot\exp\left[i\omega_0(t-\eta y)\right], \tag{S2}$$

where r_x and r_y correspond to the spot sizes of the incident pulse along x and y directions (assuming $r_y>r_x$), and ηy is the y-dependent time-delay with a proportional coefficient of η. After passing through the GTE and the optical lens, the light field at the focal plane $E_{FP}(u, v, t)$ becomes

$$E_{FP}(u,v,t)=\int_{-\infty}^{\infty}\int_{-\infty}^{\infty}E_{PFT}(x,y,t)\exp\left[i\varphi(x,y)\right]dxdy, \tag{S3a}$$

with

$$\varphi(x,y)=\phi_{GTE}(x,y)-\frac{\omega_0}{cf}(xu+yv), \tag{S3b}$$

where f is the focal length. Correspondingly, the partial derivatives of the phase $\varphi(x, y)$ to x and y can be expressed by

$$\frac{\partial\varphi(x,y)}{\partial x}=\frac{\omega_0 b}{cf}\exp\left(-\frac{x}{a}\right)\cos\left(\frac{y}{a}\right)-\frac{\omega_0 u}{cf}, \quad \text{(S4a)}$$

$$\frac{\partial\varphi(x,y)}{\partial y}=\frac{\omega_0 b}{cf}\exp\left(-\frac{x}{a}\right)\sin\left(\frac{y}{a}\right)-\frac{\omega_0 v}{cf}, \quad \text{(S4b)}$$

respectively. Here, Eqs (S4a) and (S4b) illustrate that $\varphi(x, y)$ gets the saddle points when $x=-a/2\cdot\ln[(u^2+v^2)/b^2]$ and $y=a\cdot\arctan(u/v)$. Under the stationary phase approximation [1], Eq (S3a) can be approximated as

$$E_{FP}(u,v,t)=E_{PFT}(x,y,t)\cdot\exp\left[i\varphi(x,y)\right]\cdot\frac{i2\pi\sigma}{\sqrt{\left|\frac{\partial u}{\partial x}\frac{\partial v}{\partial y}-\left(\frac{\partial u}{\partial y}\right)^2\right|}}, \quad \text{(S5)}$$

with

$$\sigma=\begin{cases}1 & for\ \ \frac{\partial u}{\partial x}\frac{\partial v}{\partial y}>\left(\frac{\partial u}{\partial y}\right)^2,\ \ \frac{\partial u}{\partial x}>0;\\ -1 & for\ \ \frac{\partial u}{\partial x}\frac{\partial v}{\partial y}>\left(\frac{\partial u}{\partial y}\right)^2,\ \ \frac{\partial u}{\partial x}<0;\\ -i & for\ \ \frac{\partial u}{\partial x}\frac{\partial v}{\partial y}<\left(\frac{\partial u}{\partial y}\right)^2.\end{cases} \quad \text{(S6)}$$

Here, $u=b\cdot\exp(-x/a)\cdot\cos(y/a)$ and $v=b\cdot\exp(-x/a)\cdot\sin(y/a)$). Accordingly,

$$\frac{\partial u}{\partial x}\frac{\partial v}{\partial y}-\left(\frac{\partial u}{\partial y}\right)^2=-\frac{b^2}{a^2}\exp\left(\frac{-2x}{a}\right)<0. \quad \text{(S7)}$$

From Eq. (S7), Eq. (S6) is simplified as $\sigma=-i$. In (u, v) coordinate system, Eq. (S5) becomes

$$E_{FP}(u,v,t)=\frac{2\pi aA}{b}\exp\left(\frac{r_x^2}{4a^2}\right)\cdot f(u,v+\eta cf)\cdot g(u,v+\eta cf,t)\cdot\exp\left[i\left(\phi(u,v+\eta cf)+\omega_0 t\right)\right], \quad \text{(S8)}$$

with

$$f(u,v)=\exp\left[-\frac{a^2}{r_x^2}\left(\ln\frac{\sqrt{u^2+v^2}}{b}+\frac{r_x^2}{2a^2}\right)^2\right]\cdot\exp\left[-\frac{a^2}{r_y^2}\arctan^2\left(\frac{v}{u}\right)\right], \quad \text{(S9a)}$$

$$g(u,v,t)=\exp\left[-\alpha\left(t-\eta a\cdot\arctan\left(\frac{v}{u}\right)\right)^2\right], \quad \text{(S9b)}$$

$$\phi(u,v) = -\frac{\omega_0 a}{cf}\left(v \arctan\left(\frac{v}{u}\right) - u \cdot \ln\left(\frac{\sqrt{u^2+v^2}}{b}\right) + u \right). \tag{S9c}$$

Eq. (S8), Eq. (S9a) and (S9b) show the phase-modulation from GTE brings $E_{FP}(u, v, t)$, the spatiotemporal coupling (STC) intensity distribution with spiral structure. However, in Eq. (S9c), the nonlinear phase term $\phi(u, v)$ presents the spiral phase distortion of the light field. To eliminate the distortion, a PCE is which possess the conjugated phase with the distorted phase term $\phi(u, v)$ is designed and set in the rear focal plane of the lens for phase compensation. In addition, in Eq. (S2), the exponential term $\exp(-i\omega_0 \eta y)$ shows the output light field has a linear phase $\omega_0 \eta y$, which results in an offset ηcf in v direction of (u, v) plane as shown in Eq. (S8). The offset stemming from the tilted pulse-front of the light field, can be easily controlled by shifting the position of the subsequent PCE. Accordingly, after the compensations of the nonlinear phase and the v-direction offset, the output light pulse becomes

$$E_{LS}(u,v,t) = \frac{2\pi a A}{b} \exp\left(\frac{r_x^2}{4a^2}\right) \cdot f(u,v) \cdot g(u,v,t) \cdot \exp(i\omega_0 t). \tag{S10}$$

Accordingly, in spectral domain it can be expressed as

$$\tilde{E}_{LS}(u,v,\omega) = \frac{2\pi a A}{b}\sqrt{\frac{\pi}{\alpha}} \exp\left(\frac{r_x^2}{4a^2}\right) \cdot f(u,v) \cdot q(u,v,\omega), \tag{S11}$$

with

$$q(u,v,\omega) = \exp\left(\frac{-(\omega-\omega_0)^2}{4\alpha}\right) \cdot \exp\left(-i(\omega-\omega_0)\eta a \cdot \arctan\left(\frac{v}{u}\right)\right). \tag{S12}$$

Eq. (S12) reveals that the compensated light field has linearly dependent spectral TC, or a spectral TC bandwidth Δl proportional to the spectral bandwidth $\Delta\omega$. If PCE is moved ed along v direction by dv, the corresponding spatial phase variation shall be

$$\begin{aligned} \Delta\phi_1(u,v) &= \phi(u,v) + \phi_{PCE}(u, v-dv) \\ &= \phi(u,v) - \phi(u, v-dv) \\ &\approx \frac{\partial \phi(u,v)}{\partial v} dv = \frac{-\omega_0 a \cdot dv}{cf} \arctan\left(\frac{v}{u}\right). \end{aligned} \tag{S13}$$

Eq. (S13) shows that the output single-coil LS carries the vortex phase in time domain with a central TC of $l_0 = -\omega_0 a \cdot dv/(cf)$, which can be tuned continuously by adjusting the

displacement dv of the PCE. As shown in Eq. (S12), the TC bandwidth Δl is independent of the PCE position, the adjustment of the central TC thus has no effects on topological charge bandwidth Δl, which means this design allows its central TC and TC bandwidth to be tuned independently.

Part II: Experimental setups

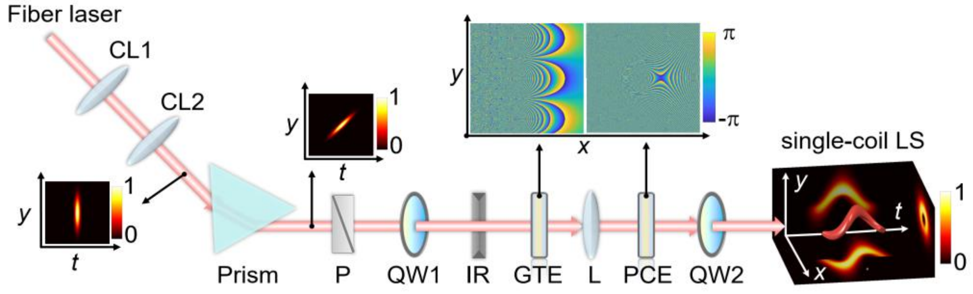

Fig. S1 Setup to generate single-coil LS. CL1 and 2: column lenses; Prism: Brewster prism; P: polarizer; QW1, 2: quarter wave plates; IR: iris; GTE: geometric transformation element; L: lens; PCE: phase compensation element. The 3D image of the single-coil LS including its projection images in the x-y, t-y and x-t planes.

The experimental setup to generate single-coil LS is shown in Fig. S1. The system mainly consists of a pulse-front tilted module and a geometric transformation module. The pulse-front tilted module comprises a Brewster prism. After passing through the Brewster prism, the incident elliptic Gaussian pulse-front (generated by a cylindrical lens pair) will be tilted along the y direction as shown in Fig. S1, resulting in different time delays at different transverse locations. The geometric transform module includes a geometric transform device GTE, a lens L, and a phase compensation device PCE. The GTE is employed to achieve log-polar Cartesian transformation, while the PCE is used to compensate the distorted phase. After passing through the geometric transform module, the pulse front-tilted pulse will be transformed to a single-coil LS due to log-polar Cartesian transformation.

The gray images of Fig. S2(a) and S2(b) depict the modulation phase distribution of GTE and PCE, respectively, under an incident light field with circular polarization. Based on the phase distribution, the corresponding devices are fabricated with liquid crystal polymer. To verify the fabricating quality, their diffraction images (red images)

have been recorded as shown by the red images in Fig. S3(a) and S3(b), which present the intensity distributions with the propagation distances of 100cm and 200cm for a linearly polarized pulsed modulated by GTE and PCE, respectively. When comparing them with the theoretical simulations, the blue images in Fig. S2(a) and S2(b), it can be inferred that they are consistent well with each other, which indicates that both the GTE and PCE have been fabricated as the required.

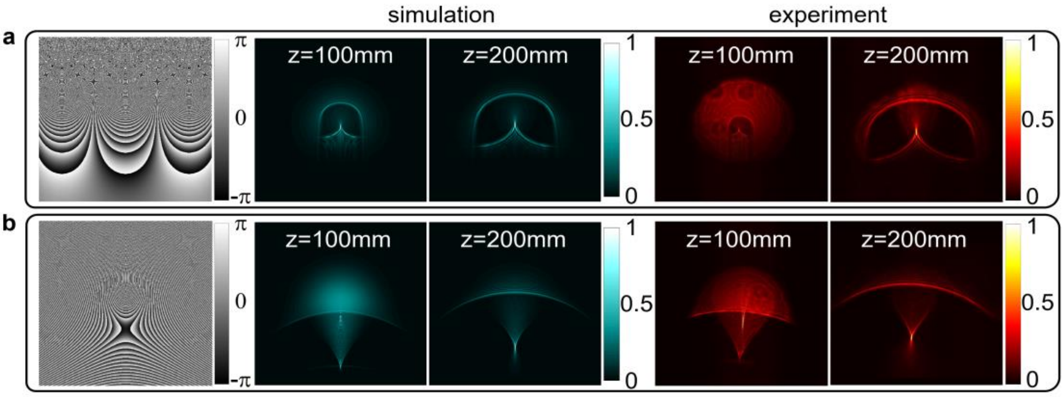

Fig. S2 The modulation phase distribution and the diffraction image of GTE and PCE. The phase modulations of GTE (**a**) and PCE (**b**): the first column (gray images) represent the simulated phase distributions, while the second and third columns show the simulated intensity profiles (blue images) of a gaussian beam after modulation, and the fourth and fifth columns (red images) are the measured intensity profiles of the laser pulse at different propagation distances from GTE or PCE.

Fig. S3 and Fig. S4 are the setups to measure the spatial-temporal intensity distribution and the spectrum-dependent TC characteristics of the single-coil LS, respectively. The measurements were taken by pump-probe method. Fig. S3 detects the sum-frequency signals between the target light field and the probe pulse at different relative time-delays with a 0.5 mm-thick type-II β-BaB_2O_4 (β-BBO) crystal for sum frequency generation. Here, Lens L1 (f =100 mm) is used to reduce the spot size of the target light field by a factor of 3.7, and to image the PCE into the β-BBO. The resultant sum-frequency signal at the β-BBO is finally imaged into a CCD camera (BASLER, acA 1600-20gm, 1626×1236) by lens L3 (f=100mm) with 1× magnification.

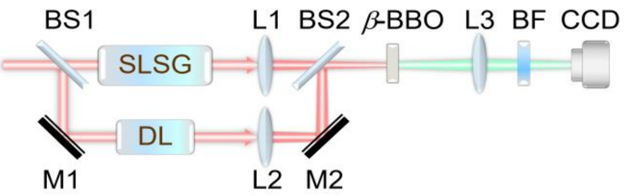

Fig. S3 Setup to detect the spatial-temporal intensity distribution of the generated single-coil LS. SLSG: generating module of single-coil LS; BS1-2: unpolarized beam splitters; DL: time-delay line; M1-2: reflectors; L1-3: lenses; β-BBO: type-II β-BaB_2O_4 crystal; BF: bandpass filter; CCD: CCD camera.

In Fig. S4, the target pulse is imaged onto the grating G1 by the 4-f imaging system including L1 and L2 with identical focal length of 125 mm, then a half waveplate (HW) is used to adjust the polarization direction of the target. A probe pulse, part of the output of the femtosecond fiber laser, has been synchronously combined with the target into G1. Here, apart from G1, lens L3, the adjustable slit (Slit), lens L4 and grating G2 form together a zero-dispersion pulse shaper. As a result, both the probe and the target are dispersed by G1, their different spectral components can thus be picked out by adjusting the position of the slit with a gap of 300 μm. After the pulse shaper, a lens L5 (f=100mm) is used to image G2 onto a CCD camera and the entrance slit of a spectrometer SP. The former records the interference images between the target and the probe, while SP captures the target spectra. Based on the interference patterns with different spectral components, the spectral TC distribution of the target pulse can be obtained.

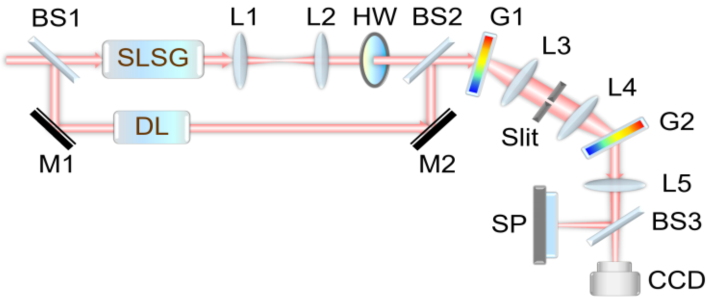

Fig. S4 Setup to detect the spectral TC value of the generated single-coil LS. SLSG: generator of the single-coil LS; BS1-3: unpolarized beam splitters; DL: time-delay line; M1-2: mirrors; L1-5: lenses; G1-2: gratings; Slit: adjustable Slit; SP: spectrometer; CCD: CCD camera.

Part III: Supplementary Experimental Results

Figs. S5~S7 present the measurements of the spectral TC values with different

central TCs selected by l_0=2, 0 or −3, respectively. The measurements were taken by recording the interference patterns of the single-coil LS and the probe beams at different narrowband spectra, which are obtained with the slit of Fig. S4. When moving the slit, the central wavelengths of the narrowband spectra change from 1023nm to 1039nm. Figs. S5(m), S6(m) and S7(m) correspond to l_0=2, 0 and −3, where the average bandwidths of the narrowband spectra are 1.30nm, 1.28nm and 1.24nm, and the intervals between adjacent narrowband spectra are 1.48nm, 1.42nm and1.46nm, respectively. Figs. S5(a-l)~S7(a-l) present the measured interference patterns, from which, the extracted spectral TC values of the single-coil LSs varies from 7 to −4 for l_0=2, from 5 to −6 for l_0=0, and from 2 to −9 for l_0=−3, respectively.

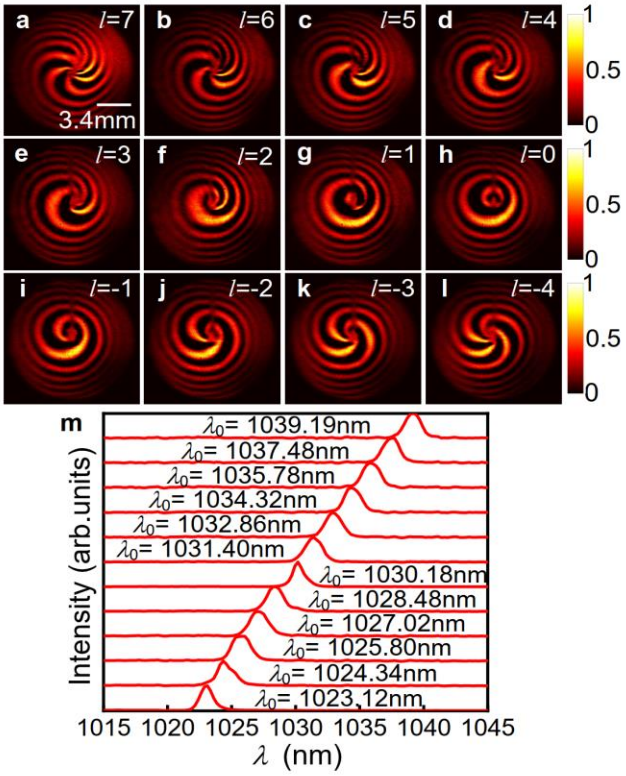

Fig. S5 The single-coil LS interference patterns with a central TC value l_0=2. a–l The interference patterns between the output pulse and the femtosecond probes with the selected central wavelength. **m** The probe pulse at different narrowband spectra.

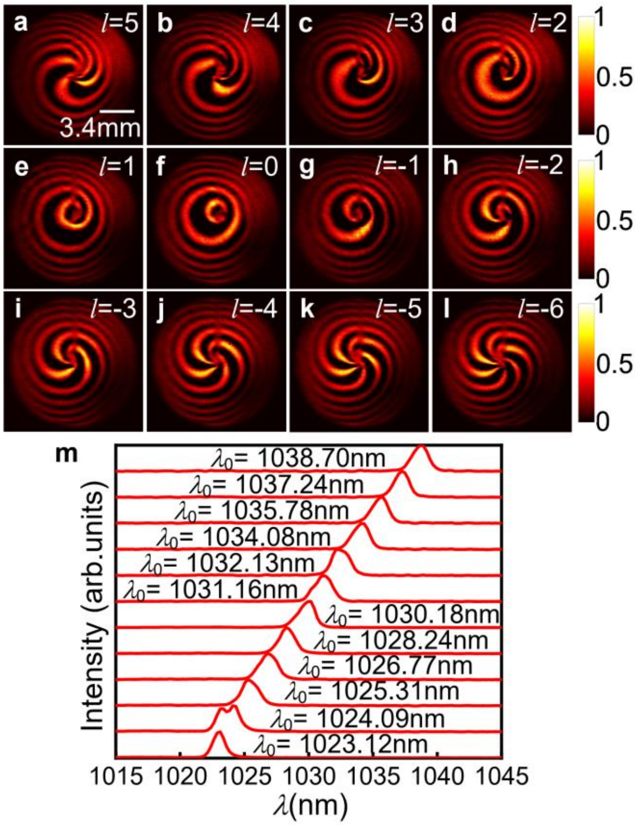

Fig. S6 The single-coil LS interference patterns with a central TC value *l_0*=0. a–l The interference patterns between the output pulse and the femtosecond probes with the selected central wavelength. **m** The probe pulse at different narrowband spectra.

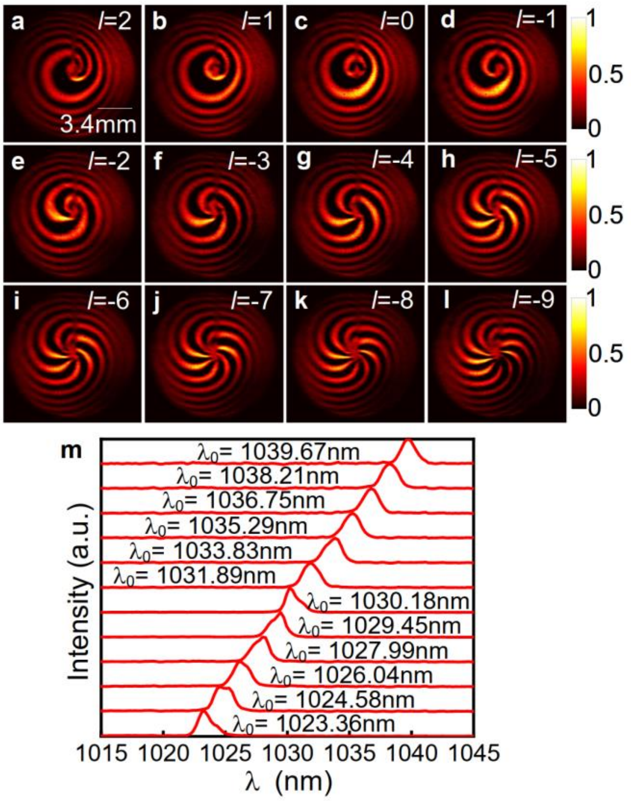

Fig. S7 The single-coil LS interference patterns with a central TC value l_0=–3. a–l The interference patterns between the output pulse and the femtosecond probes with the selected central wavelength. **m** The probe pulse at different narrowband spectra.

Part IV: Simulation examples to generate others spatiotemporal coupling light fields

1. Multi-coil light spring

With the proposed technique, the multi-coil light spring can be realized by serval different approaches including introducing a pulse train incidence, increasing the length of the incident long elliptical pulse, or reducing the size coefficient of the geometric transformation device (GTE) and phase compensation device (PCE). Here we take changing the size coefficient of GTE and PCE as an example, such approach does not require different laser source or other elements. In the log-polar-to-Cartesian transformation, the coordinates (x, y) and (u, v) satisfies the following relationship

$$u = b \cdot \exp\left(-\frac{x}{a}\right) \cdot \cos\left(\frac{y}{a}\right), \tag{S14a}$$

$$v = b \cdot \exp\left(-\frac{x}{a}\right) \cdot \sin\left(\frac{y}{a}\right). \tag{S14b}$$

According to Eq. (S14a) and (S14b), both u and v changes periodically when y increases. Therefore, when the incident pulse has a dimension of $2N\pi a$ (N: a positive integer) along the y direction, it produces a light spring with n rotation periods. Therefore, by substituting "a" in Eq. (S1a) and (S1b) with "a/N", a multi-coil light spring with N rotational cycles can be generated. Fig. S8(a) and S8(b) show the simulated spatiotemporal intensity structure of LSs with 2 and 3 rotation periods, respectively. The parameters set for the simulation are as follows: a=0.9mm (two-coil LS) and 0.6mm (three-coil LS), b=2.4 mm, f=500mm. The seed is a 172fs -1030nm pulse with the transverse sizes of 11.3mm × 0.45mm (FWHM). The maximal delay of the pulse-front tilt is 2.51ps.

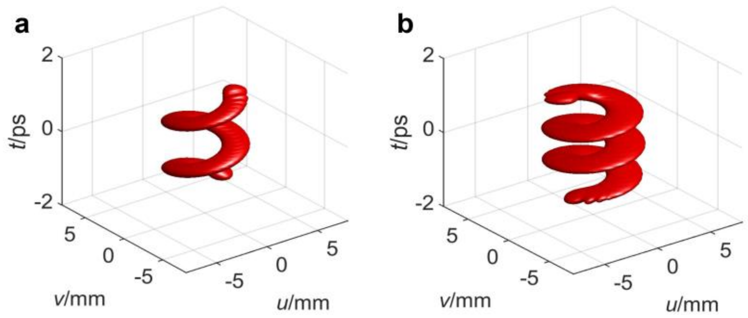

Fig. S8 Simulation results of multi-coil light springs. a Two-coil LS and **b** three-coil LS.

2. Rotating light field with the 8-shape trajectory

By modifying geometric transformation relationships, this design may also control the beam rotation trajectory over time. In this example, we generate a rotating optical field with the 8-shape trajectory, where the coordinates (x, y) and (u, v) before and after the geometric transformation satisfy the following relationship

$$u = a \cdot \exp\left(\frac{x}{b}\right) \cdot \left(\cos\left(\frac{y}{c}\right)\right)^{-\frac{c}{b}}, \quad \text{(S15a)}$$

$$v = a \cdot \exp\left(\frac{x}{b}\right) \cdot \sin\left(\frac{y}{c}\right) \cdot \left(\cos\left(\frac{y}{c}\right)\right)^{-\frac{b+c}{b}}, \quad \text{(S15b)}$$

where a is the scale factor, while b and c are the structure coefficients used to adjust geometric transformation (a>0, b<0, c>0). Based on the transformation relationship, we can derive the corresponding phase distribution of the geometric transformation device (GTE) and phase compensation device (PCE)

$$\phi_{GTE}(x,y)=\frac{kab}{f}\cdot\exp\left(\frac{x}{b}\right)\cdot\left(\cos\left(\frac{y}{c}\right)\right)^{-\frac{c}{b}}, \tag{S16a}$$

$$\phi_{PCE}(u,v)=\frac{k}{f}\left\{c\cdot v\cdot\arctan\left(\frac{v}{u}\right)+b\cdot u\cdot\left[\ln\left(\frac{|u|}{a}\left(1+\frac{v^2}{u^2}\right)^{-\frac{c}{2b}}\right)-1\right]\right\}, \tag{S16b}$$

where k is the wave vector, and f is the lens focal length. Assuming that the incident pulse with pulse-front tilt is

$$E_{PFT}(x,y,t)=A\cdot\exp\left(-\frac{x^2}{r_x^2}-\frac{y^2}{r_y^2}\right)\cdot\exp\left[-\alpha(t-\eta y)^2\right]\cdot\exp(i\omega_0 t), \tag{S17}$$

With A represents the amplitude, α is pulse width parameter, and η is the pulse-front tilt coefficient. r_x and r_y stand for the beam sizes along the x and y directions. Since the influence of the transverse phase introduced by the pulse-front tilt is easy to be eliminated, the transverse phase of the pulse-front tilt pulse can be ignored in Eq. (S17). Under the stable phase approximation, the corresponding light field distribution can be approximated as

$$E_{RLF}(u,v,t)=f_{RLF}(u,v)\cdot g_{RLF}(u,v,t)\cdot\exp(i\omega_0 t), \tag{S18a}$$

$$f_{RLF}(u,v)=\frac{2\pi Af}{k^2}\cdot\sqrt{\frac{-bc}{u^2+v^2}}\cdot\exp\left[-\frac{c^2}{r_y^2}\arctan^2\left(\frac{v}{u}\right)-\frac{b^2}{r_x^2}\ln^2\left(\frac{|u|}{a}\cdot\left(1+\frac{v^2}{u^2}\right)^{-\frac{c}{2b}}\right)\right], \tag{S18b}$$

$$g_{RLF}(u,v,t)=\exp\left[-\alpha\cdot\left(t-c\eta\cdot\arctan\left(\frac{v}{u}\right)\right)^2\right]. \tag{S18c}$$

Here, $f_{RLF}(u, v)$ describes the output pulse as the motion trajectory with an 8-shape, while $g_{RLF}(u, v, t)$ demonstrates that it has angular delay linearly. However, due to the non-circular motion path of the output light field, the linear angular delay induces variable speed rotational behavior. Fig. S9 depicts a simulated rotating light field with the 8-shape trajectory obtained through angle-spectral diffraction. The parameters set for the simulation are as follows: a=3mm, b= −1.7mm, c=1.59mm, and f=500mm. The seed pulse is the same as that used for Fig. 8S, the maximal delay of the pulse-front tilt is thus 2.51ps as well.

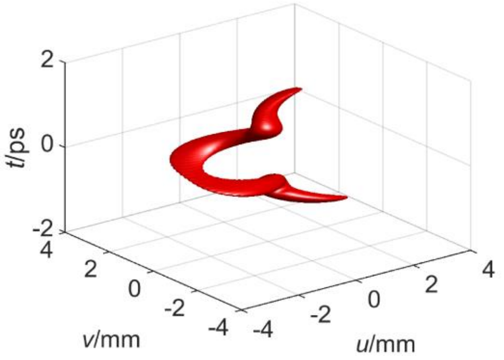

Fig. S9 Simulation results of the rotating light field with the 8-shope trajectory. The light spot rotates counterclockwise in an 8-shope trajectory with time.

3. Annular space-time light field with the mixed polarization

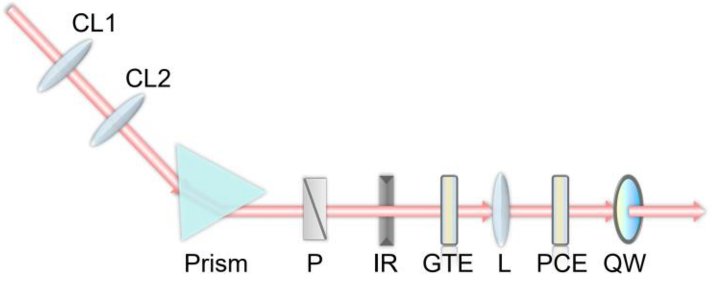

Fig. S10 Setup to generate the annular space-time coupled light field. CL1 and 2: column lenses; Prism: Brewster prism; P: polarizer; IR: iris; GTE: geometric transformation element; L: lens; PCE: phase compensation element; QW: quarter wave plates.

Liquid crystal can be used to introduce opposite phase modulations for two beams with conjugately circular polarizations, which allows to generate a STC light field with the mixed polarization. The corresponding experimental setup is illustrated as Fig. S10 (QW1 has been removed compared to Fig. S1). In this example, we will present the principle and simulation results of generating annular space-time coupled light with mixed polarization.

Assuming the incident pulse is linearly polarized with a pulse-front tilt, i.e.

$$E_{PFT}(x,t) = A\cdot\exp\left(-\frac{x^2}{r_0^2}\right)\cdot\exp\left[-\alpha(t-\eta x)^2\right]\cdot\exp(i\omega_0 t), \tag{S19}$$

Where A, α and η are the parameters as described for Eq. (S17). r_0 is the spot size of the pulse. In this example, the evolution of the light field mainly takes place on the x-t

coordinate, so the influence of the *y* coordinate is ignorable. As in the previous example, we also neglected the transverse phase introduced by the pulse-front tilt.

Here, the geometric transform system is composed of the geometric transform device GTE, the cylindrical lens *L*, the phase compensation device PCE, and a quarter wave plate QW. Assuming that GTE and PCE are made of liquid crystal materials, the phase distributions of GTE and PCE for right-handed circularly polarized light can be expressed as

$$\phi_{GTE}(x)=\frac{ak}{2f}\left(|x|\cdot\sqrt{b^2-x^2}+b^2\cdot\arctan\left(\frac{|x|}{\sqrt{b^2-x^2}}\right)\right), \tag{S20a}$$

$$\phi_{PCE}(u)=-\frac{k}{2f}\left(\frac{|u|\cdot\sqrt{a^2b^2-u^2}}{a}-ab^2\cdot\operatorname{arccot}\left(\frac{|u|}{\sqrt{a^2b^2-u^2}}\right)\right), \tag{S20b}$$

where, *a* and *b* are the size coefficients, *k* is the wave vector, *f* is the focal of the cylindrical lens. For the left-circularly polarized light, the introduced phases are $-\phi_{GTE}$ and $-\phi_{PCE}$, respectively. When the linearly polarized pulse with a pulse front-tilted passes through the geometric transformation system, due to the properties of liquid crystal, different phase will be added to different circular polarization components, which makes the final output light field an annular space-time light field containing two polarization states. Under the stable phase approximation, the corresponding light field distribution can be approximated as

$$E_{out}(u)=f(u)\cdot g(u)\cdot\exp(i\omega_0 t), \tag{S21a}$$

with

$$f(u)=A\sqrt{\frac{\pi f}{2ak}}\cdot\frac{\sqrt{|u|}}{\left(a^2b^2-u^2\right)^{1/4}}\cdot\exp\left(-\frac{a^2b^2-u^2}{a^2{r_0}^2}\right), \tag{S21b}$$

$$\begin{aligned}g(u)=&\exp\left[-\alpha\left(t-sign(u)\cdot\frac{\eta}{a}\sqrt{a^2b^2-u^2}\right)^2-\frac{\pi}{4}i\right]\boldsymbol{e}_{\parallel}\\&+\exp\left[-\alpha\left(t+sign(u)\cdot\frac{\eta}{a}\sqrt{a^2b^2-u^2}\right)^2+\frac{3\pi}{4}i\right]\boldsymbol{e}_{\perp},\end{aligned} \tag{S21c}$$

where $\boldsymbol{e}_{\parallel}$ and $\boldsymbol{e}_{\perp}$ are the unit vectors of horizontal and vertical directions, which are used to describe the polarization direction of the pulse, and *sign*(*u*) is the symbolic function. According to Eq. (S21c), it can be observed that the output light field possesses a

toroidal angular delay, which means that the output light field has a toroidal spatiotemporal structure. Accordingly, based on the theoretical analysis above, the proposed mechanism can also generate a toroidal space-time light field by the special design. Fig. S11 shows spatiotemporal intensity structure of the output light field based on the simulation of the diffraction propagation. And the red and blue part correspond to light field with horizontal polarization, and vertical polarization, respectively. The spatiotemporal structure shown in the figure is consistent with the theoretical predictions of Eq. (S21a-S21c). The parameters set by the simulation are as follows: a=0.3, b=5.03mm, f=500mm. The pulse with a pulse-front tilt has a pulse width of 172fs, a central wavelength of 1030nm, and a pulse size of 10.1mm and 0.4mm (FWHM). The maximal delay of the pulse-front tilt is 2ps.

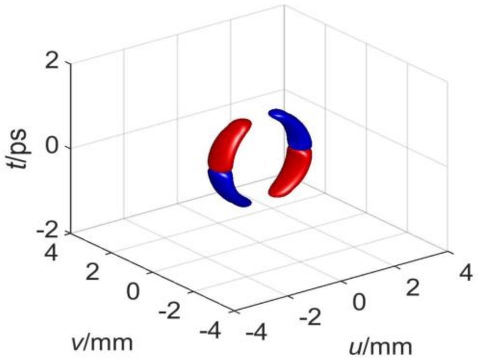

Fig. S11 Simulation results of annular space-time light field based on diffraction propagation. The red and blue correspond to horizontal polarization, and vertical polarization, respectively.

Part IV: Supplementary References

1. W. J. Hossack, A. M. Darling and A. Dahdouh, "Coordinate Transformations with Multiple Computer-generated Optical Elements," J. Mod. Optic. **34**, 1235-1250 (1987).
2. G. C. G. Berkhout, M. P. J. Lavery, J. Courtial, M. W. Beijersbergen, and M. J. Padgett, "Efficient sorting of orbital angular momentum states of light," Phys. Rev. Lett. **105**, 153601 (2010).
